# Complete mitochondrial genome of the important phytopathogenic fungus *Macrophomina phaseolina* (Botryosphaeriales, Ascomycota)

**DOI:** 10.1080/23802359.2021.1975505

**Published:** 2021-09-17

**Authors:** Haitian Yu, Liping Wang, Feng Yang, Yuhua He, Meiyuan Lv

**Affiliations:** Institute of Food Crops, Yunnan Academy of Agricultural Sciences, Yunnan, China

**Keywords:** *Macrophomina phaseolina*, mitochondrial genome, phylogenetic analyses

## Abstract

*Macrophomina phaseolina* is a Catastrophic plant pathogen, which can cause serious reduction in crop production. In the current study, the mitochondrial genome of *M. phaseolina* is assembled and annotated. The mitogenome of *M. phaseolina* is a circular molecule of 101,198 bp. The overall nucleotide content is 34.95% A, 35.25% T, 13.30% C, 16.50% G, with a CG content of 29.80%. The mitogenome contains 42 genes, including 14 protein coding genes (PCGs), 2 ribosomal RNA (rRNA) genes and 26 transfer RNA (tRNA) genes. Phylogenetic analyses based on concatenated protein sequences from 15 taxa of five orders in Ascomycota indicated that *M. phaseolina* is clustered in the order Botryosphaeriales. This study would have a positive impact on the molecular biology research and biological control of *Macrophomina* fungi in the future.

*Macrophomina phaseolina* (Tassi) Goid., the type of the *Macrophomina*, belongs to order Botryosphaeriaceae (Botryosphaeriales, Ascomycota) (Crous et al. 2006; Phillips et al. 2013; Ghosh et al. 2018), is the most common species of the genus and synonymous with *Tiarosporella phaseolina* (Tassi) Vander Aa. In 1982, Goidanich named it *Macrophomina phaseolina* (Tassi) Goid, and this name is still in use today (Ghosh et al. 2018). *M. phaseolina* is an important soilborne plant pathogenic fungus that can cause charcoal rot disease on about 500 plant species of more than 100 families all over the world and can cause significant yield losses (Suriachandraselvan et al. 2005). The fungus can survive in soil for up to 15 years (Su et al. 2001; Babu et al. 2007; Ghosh et al. 2018). Soybean carbon rot caused by *M. phaseolina* is one the most serious diseases that can reduce the yield of soybean in the world. Under the conditions of heat and drought stress, soybean carbon rot caused up to 77% plant mortality, which seriously affected soybean yield and quality (Allen et al. 2017). Sun et al. (2019) first reported identification of charcoal rot caused by *M. phaseolina* on faba bean in China. This disease caused serious yield loss of faba bean, infected plants initially showed leaf chlorosis and wilting, and the plants eventually died with the leaves remaining attached. The identification of the phylogenetic position and the study of genetic diversity of *M. phaseolina* are conducive to the prevention and control of charcoal rot. However, our understanding of the genetic diversity and phylogenetic position based on mitochondrial genome (mitogenome) of *M. phaseolina* is lacking. The current study we first reported the complete mitogenome of *M. phaseolina* and determine its systematic position, which would have a positive impact on the molecular biology research and biological control of *Macrophomina* fungi in the future.

In this study, the *M. phaseolina* strain was isolated from the diseased root of *Vicia faba* L. collected in Fumin County, Yunnan, China (25°18′40″N, 103°07′25″E, alt. 1903 m). A specimen was deposited in the herbarium at Yunnan Academy of Agricultural Sciences (http://www.yaas.org.cn/, Haitian Yu, email: haitianlegume@outlook.com) under the voucher number YN23. Mycelia cultured on PDA at 20 °C for 2 weeks were used to extract total genomic DNA using MiniBEST Plant Genomic DNA Extraction Kit (TaKaRa, China). To validate the DNA integrity and quality, electrophoresis on 1.0% agarose gels was used. The purified DNA was sequenced by the Illumina sequencing platform (HiSeq PE150). Mitogenomic sequences of high quality reads were assembled by employing the programmes the SPAdes 3.9.0 with default parameterbe (Bankevich et al. 2012). The mitogenome annotation were conducted with MFannot tool and ARWEN web server, combined with manual corrections. The mitogenomic circular map of *M. phaseolina* was drawn by Organellar Genome DRAW tool (Lohse et al. 2007).

The complete mitogenome of *M. phaseolina* (GenBank accession: MT MW557546) is a circular molecule of 101,198 bp. The overall nucleotide content is 34.95% A, 35.25% T, 13.30% C, 16.50% G, with a CG content of 29.80%. The mitogenome contains 42 genes, including 14 protein coding genes (PCGs), 2 ribosomal RNA (rRNA) genes and 26 transfer RNA (tRNA) genes. Protein-encoding genes include two ATP synthase subunits (*atp6* and *atp9*), three cytochrome oxidase subunits (*cox1*, *cox2*, and *cox3*), one apocytochrome b (*cob*), seven NADH dehydrogenase subunits (*nad1*, *nad2*, *nad3*, *nad4*, *nad4L*, *nad5*, and *nad6*) and one ribosomal protein (*rps3*).

To verify the systematic position of *M. phaseolina*, mitogenomic sequences of 14 species in Ascomycota downloaded from NCBI were employed. Sequence alignment and phylogenetic analysis were conducted by complying with methods of Wang et al. (2020) and Wang et al.(2021). Fifteen concatenated mitochondrial PCGs were employed for phylogenetic analysis. Phylogenetic analysis was carried out using Maximum Likelihood (ML) method with RaxML 7.0.3 (Stamatakis 2006). The phylogenetic tree is composed of 5 orders (Figure 1), viz. Pleosporales, Botryosphaeriales, Dothideales, Cladosporiales and Mycosphaerellales. Phylogenetically, *M. phaseolina* is clustered in the order Botryosphaeriales with a high credible support by BI posterior probabilities (PP = 100%).

**Figure 1.**
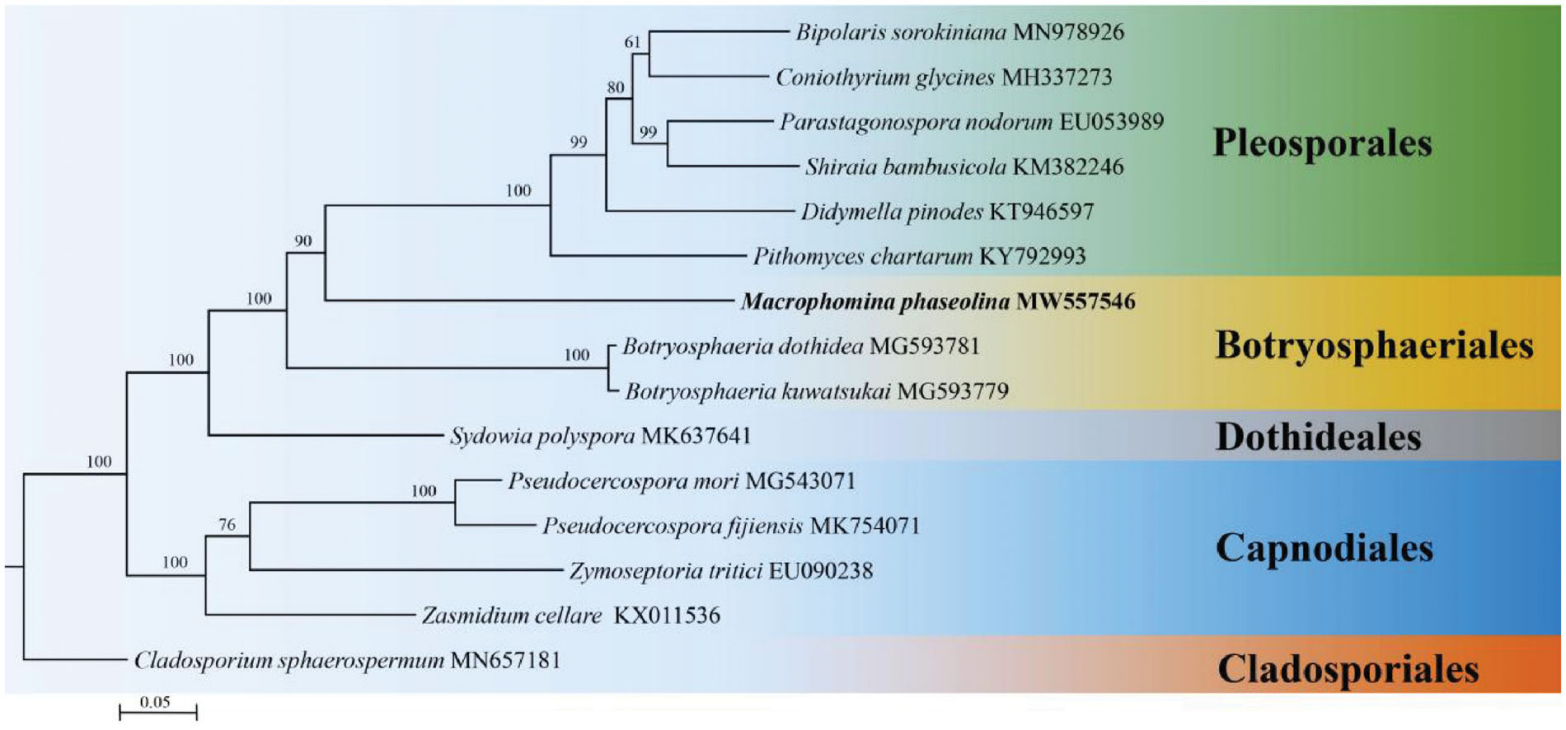
Phylogenetic relationships among 15 species based on Maximum likelihood (ML) analysis from 15 concatenated mitochondrial protein-coding genes (PCGs). The 15 PCGs include subunits of the respiratory chain complexes (*cob*, *cox1*, *cox2*, *cox3*), ATPase subunits (*atp6*, *atp8*, *atp9*), NADH: quinone reductase subunits (*nad1*, *nad2*, *nad3*, *nad4*, *nad4L*, *nad5*, *nad6*) and one ribosomal protein (*rps3*).

## Data Availability

This mitogenome of *Macrophomina phaseolina* was submitted to GenBank under the accession number of MW557546 (https://www.ncbi.nlm.nih.gov/nuccore/MW557546). The associated BioProject, SRA, and Bio-Sample numbers are PRJNA751633, SRR15329826, and SAMN20524426, respectively.
